# Comparison of Database Search Methods for the Detection of *Legionella pneumophila* in Water Samples Using Metagenomic Analysis

**DOI:** 10.3389/fmicb.2018.01272

**Published:** 2018-06-19

**Authors:** Jednipit Borthong, Ryosuke Omori, Chihiro Sugimoto, Orasa Suthienkul, Ryo Nakao, Kimihito Ito

**Affiliations:** ^1^Division of Bioinformatics, Research Center for Zoonosis Control, Hokkaido University, Sapporo, Japan; ^2^Precursory Research for Embryonic Science and Technology, Japan Science and Technology Agency, Kawaguchi, Japan; ^3^Division of Collaboration and Education, Research Center for Zoonosis Control, Hokkaido University, Sapporo, Japan; ^4^Global Institute for Collaborative Research and Education, Hokkaido University, Sapporo, Japan; ^5^Faculty of Public Health, Thammasat University, Rangsit Campus, Pathumthani, Thailand; ^6^Laboratory of Parasitology, Graduate School of Veterinary Medicine, Hokkaido University, Sapporo, Japan

**Keywords:** water-borne diseases, metagenomic analysis, bacteria, detection, receiver operating characteristic curve, *Legionella pneumophila*

## Abstract

Metagenomic analysis has become a powerful tool to analyze bacterial communities in environmental samples. However, the detection of a specific bacterial species using metagenomic analysis remains difficult due to false positive detections of sequences shared between different bacterial species. In this study, 16S rRNA amplicon and shotgun metagenomic analyses were conducted on samples collected along a stream and ponds in the campus of Hokkaido University. We compared different database search methods for bacterial detection by focusing on *Legionella pneumophila*. In this study, we used *L. pneumophila*-specific nested PCR as a gold standard to evaluate the results of the metagenomic analysis. Comparison with the results from *L. pneumophila*-specific nested PCR indicated that a blastn search of shotgun reads against the NCBI-NT database led to false positive results and had problems with specificity. We also found that a blastn search of shotgun reads against a database of the catalase-peroxidase (*katB*) gene detected *L. pneumophila* with the highest area under the receiver operating characteristic curve among the tested search methods; indicating that a blastn search against the *katB* gene database had better diagnostic ability than searches against other databases. Our results suggest that sequence searches targeting long genes specifically associated with the bacterial species of interest is a prerequisite to detecting the bacterial species in environmental samples using metagenomic analyses.

## Introduction

Metagenomic analysis has become a powerful tool for analyzing bacterial communities in environmental samples. In metagenomic analyses, genetic materials in samples are analyzed directly by next generation sequencing (NGS) (Thomas et al., 2012). In contrast to single gene amplification techniques such as PCR-based assays, metagenomic analysis can detect genomic fragments of thousands of bacteria in a single NGS run (Caporaso et al., 2012). Metagenomic approaches have been used to investigate the bacterial population structure in a variety of samples, including environmental (Daniel, 2005; Sogin et al., 2006; Breitbart et al., 2009), food (Ercolini, 2013), and clinical samples (Cho and Blaser, 2012). The price of NGS platforms and their running costs are decreasing (Lecuit and Eloit, 2014; Muir et al., 2016), increasing the opportunity for application in metagenomic analysis (Garrido-Cardenas and Manzano-Agugliaro, 2017).

Several studies have evaluated the diagnostic potential of metagenomic analysis in clinical settings. Nakamura et al. detected genomic fragments of *Campylobacter jejuni* from the fecal sample of a diarrheal patient using metagenomic analysis (Nakamura et al., 2008). During an outbreak of acute respiratory distress syndrome in Germany in 2013, Fischer et al. (2014) used metagenomic analysis on patient bronchoalveolar lavage samples to confirm that *Chlamydia psittaci* was the causative agent of the outbreak. Ortiz-Alcantara et al. (2016) conducted metagenomics analysis using cerebrospinal fluid of a pediatric patient with meningitis to identify the causative agent as *Psychrobacter* sp. Kujiraoka et al. (2017) showed that metagenomic analysis was useful for rapid bacterial diagnosis of acute cholecystitis. These studies suggest that metagenomic analysis can be used for the diagnosis of infectious diseases when routine methods fail to detect pathogens.

Early detection of potential pathogens in the environment is one of the most important strategies to prevent waterborne and foodborne infectious diseases (Pandey et al., 2014). There are two major approaches in pathogen detection with metagenomic analysis. The first approach, 16S rRNA metagenomic analysis, uses conserved and variable regions in the bacterial 16S rRNA gene to study the taxonomy of bacteria in samples (Janda and Abbott, 2007). 16S rRNA metagenomic analysis has been used to detect pathogens in water and food in numerous studies. Ibekwe et al. detected potential pathogens from the genera *Aeromonas, Clostridium, Bacillus, Pseudomonas*, and *Treponema* in water samples collected from the Middle Santa Ana River (Ibekwe et al., 2013). Ye and Zhang detected pathogens from wastewater treatment plants in China, United States, Canada, and Singapore; finding all samples contaminated with *Aeromonas* and *Clostridium* (Ye and Zhang, 2011). Mukherjee et al. (2016) investigated the bacterial diversity in water supplies from rural areas in Haiti and found human pathogens such as *Aeromonas, Bacillus, Clostridium*, and *Yersinia* in a high proportion of bacterial communities. Several studies applied 16S rRNA analysis to check pathogen contamination in drinking water (Shi et al., 2013; Huang et al., 2014; Pinto et al., 2016; Oh et al., 2018) and vegetables (Leonard et al., 2015; Kim et al., 2018). 16S rRNA metagenomic analysis uses PCR, and the results are affected by this amplification step. Problems due to differences in the copy number of 16S rRNA gene in a genome of bacteria (Vetrovsky and Baldrian, 2013) and chimeric sequences in PCR products (Haas et al., 2011) may arise. No single hypervariable region can be used to differentiate between bacteria (Chakravorty et al., 2007), and closely related bacteria cannot be differentiated (Weinstock, 2012).

The second approach is shotgun metagenomic analysis. By using random primers, DNA fragments can be captured from any part of the bacterial genome (Sharpton, 2014). Since the bacterial genome contains sequences specific to a bacterial species, there is a possibility to increase the specificity of pathogen detection. Several studies have applied whole genome metagenomics to the detection of potential pathogens in the environment. Lu et al. (2015) compared bacterial populations in water before and after processing in a sewage treatment system, and they found that most pathogenic bacteria were eliminated after the treatment. Nordahl Petersen et al. (2015) investigated toilet waste from airplanes using metagenomics and detected *Salmonella enterica* and *Clostridium difficile* from the waste after international flights. Several other studies have used whole genome metagenomics to investigate pathogenic bacteria in water samples collected from wastewater treatment (Cai and Zhang, 2013; Ibarbalz et al., 2016), drinking water and drink water systems (Gomez-Alvarez et al., 2012; Chao et al., 2013; Otten et al., 2016), and freshwater (Van Rossum et al., 2015; Mohiuddin et al., 2017). Whole genome metagenomics are used for food safety (Walsh et al., 2017) and investigation of the food production chain (Yang et al., 2016). These studies show the potential usefulness of metagenomic analyses in detecting pathogenic bacteria in environmental samples. Shotgun metagenomics can get narrower sequence coverage than 16S rRNA analysis (Angiuoli et al., 2011). The bacterial diversities analyzed by shotgun metagenomics depend on the method of DNA extraction and/or sequencing protocol (Morgan et al., 2010) and can also capture the host’s genetic material (Kuczynski et al., 2011).

Taxonomic classification is a bioinformatics procedure to infer the population structure of microorganisms based on genomic information obtained from samples, and several computational methods have been developed so far (Lindgreen et al., 2016). The lowest common ancestor (LCA) algorithm implemented in MEGAN assigns sequence reads to taxa on taxonomical trees based on blastn search results of reads against given databases (Huson et al., 2007). Kraken (Wood and Salzberg, 2014), CLARK (Ounit et al., 2015), and One Codex (Minot et al., 2015) use the differences in *k*-mer distributions among taxa to assign reads to nodes in the taxonomic tree. MetaPhlAn2 uses pre-defined sets of clade-specific marker sequences and classifies reads using reference mapping onto marker sequences (Truong et al., 2015). MGmapper uses alignment scores from reference mappings of reads to reference sequences in a database (Petersen et al., 2017). RDP (Cole et al., 2005) and SILVA (Quast et al., 2013) are specialized to analyze 16S rRNA amplicon reads and determine the taxa of reads according to sequence similarity of the 16S rRNA genes.

Despite recent advancements in sequencing technologies and classification algorithms, several studies using metagenomic analyses have exposed important issues associated with sensitivity and specificity. Loman et al. (2013) reported false negative detections of Shiga-Toxigenic *Escherichia coli* O104:H4 in the diagnosis of diarrheal patients using metagenomic analysis. Several groups found that bacterial populations identified by 16S rRNA metagenomics and those by shotgun metagenomics were not always consistent with one another (Shah et al., 2011; Clooney et al., 2016). These results suggest that sensitivity and/or specificity of the two methods are different depending on the bacterial species. It is also known that metagenomic analyses generate different results depending on the taxonomical classification algorithms (Clooney et al., 2016) and reference databases (Miller et al., 2013) used.

*Legionella pneumophila* is the causative agent of Legionnaire’s diseases. This pathogenic bacterium is ubiquitous in natural aquatic environments such as ponds, lakes, rivers, and estuaries (Fliermans et al., 1981). *L. pneumophila* can be also found in man-made water reservoirs, such as cooling towers (Turetgen et al., 2005), spas (Benkel et al., 2000), and water distribution systems (Stout et al., 1985). Inhalation of water aerosols is the primary cause of transmission to humans, and human-to-human transmission is rare (Correia et al., 2016).

The standard methods of detecting *L. pneumophila* in water samples are the culture-based and PCR-based methods. The cultured-based method uses centrifugation, filtration, heat and acid treatments, selective media, and antibiotics (Atlas et al., 1995). This method can be used to enumerate the total population of *L. pneumophila* in samples. The nested PCR and real-time PCR are alternative assays for the detection of *L. pneumophila*. These PCR-based methods use the primer sequences of the genes specific to *L. pneumophila*. The 5S rRNA (Mahbubani et al., 1990), 16S rRNA (Cloud et al., 2000; Buchbinder et al., 2002), *dotA* (Yanez et al., 2005), and *mip* (Mahbubani et al., 1990; Catalan et al., 1994) are examples of target genes for the detection of *L. pneumophila*.

Several metagenomic studies detected *Legionella* spp. and *L. pneumophila* in water samples (Cai and Zhang, 2013; Delafont et al., 2013; Lu et al., 2015; Mohiuddin et al., 2017). Pereira et al. (2017) conducted 16S rRNA metagenomic analysis and detected six different *Legionella* spp. in freshwater samples. Peabody et al. (2017) investigated *Legionella* spp. in water samples from seven different places for a year. They found that *L*. pneumophila was the most abundant at all sampling sites (Peabody et al., 2017).

Sequence-based typing (SBT) and core genome multilocus sequence typing (cgMLST) are used for outbreak investigation of Legionnaires’ disease (Gaia et al., 2003; Moran-Gilad et al., 2015). Both methods use nucleotide sequences at seven alleles on the genome of *L. pneumophila* to determine sequence type (Gaia et al., 2005; Ratzow et al., 2007). Whole genome sequencing (WGS) has become a tool for differentiation among *L. pneumophila* (Reuter et al., 2013; Graham et al., 2014; Levesque et al., 2014; McAdam et al., 2014). WGS maps the NGS reads onto the reference sequences and analyzes single nucleotide polymorphism in the genome. The cgMLST uses more than 1,500 loci in core genes of *L. pneumophila* (Moran-Gilad et al., 2015; Burckhardt et al., 2016; Petzold et al., 2017).

The aim of this study is to compare different database search methods for detecting *L. pneumophila* in metagenomic analyses. Using water samples collected from a stream and ponds in the campus of Hokkaido University, 16S rRNA and shotgun metagenomic analyses were conducted. In this study, we used *L. pneumophila*-specific nested PCR as a gold standard to evaluate the results of the metagenomic analysis.

## Materials and Methods

### Water Samples

**FIGURE 1:**
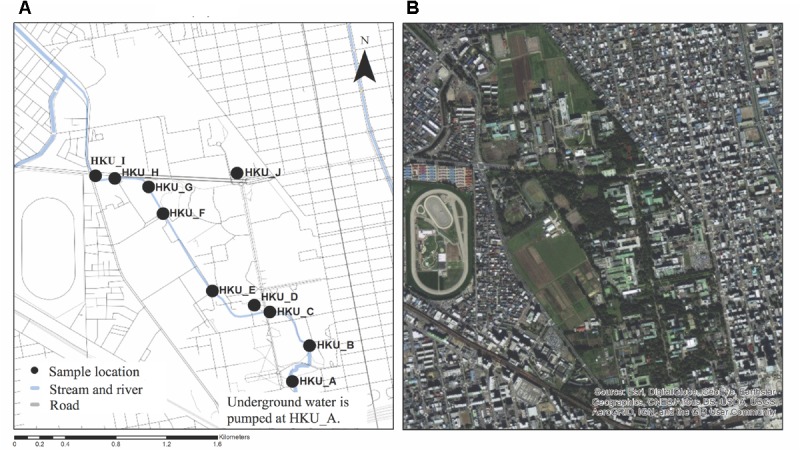
Locations of sample collection. Water samples were collected from Sakushukotoni stream (HKU_A, HKU_B, HKU_C, HKU_E, HKU_F, HKU_G, HKU_H, and HKU_I), Ohno Pond (HKU_D), and Hyotan Pond (HKU_J). **(A)** A map from OpenStreetmap. **(B)** A satellite image from Google Earth.

Ten water samples were collected in the Sapporo campus of Hokkaido University on October 16th, 2012. Eight samples were obtained from different points along the Sakushukotoni stream (HKU_A, HKU_B, HKU_C, HKU_E, HKU_F, HKU_G, HKU_H, and HKU_I), one sample was collected from Ohno Pond (HKU_D), and another sample was collected from Hyotan Pond (HKU_J) (**Figure 1**). Two liters of water were collected from the water surface using sterilized containers (Pope and Patel, 2008; Tekera et al., 2011; Silva et al., 2012). The samples were transferred to a laboratory of the Research Center for Zoonosis Control in Hokkaido University for further analysis.

### Bacterial Concentration and DNA Extraction

Bacteria in the water samples were concentrated using a standard membrane filtration technique with four different pore sizes; 100, 10, 5, and 0.22 μm (Millipore, Tokyo, Japan). The filtrates of 0.22 μm-membrane were used to extract DNA using a PowerWater^®^ DNA Isolation Kit (Mo Bio Laboratories, Inc., Carlsbad, CA, United States). DNA concentration was determined using a Qubit^TM^ fluorometer (Invitrogen, Tokyo, Japan).

### Detection of *Legionella* spp. and *L. pneumophila* Using Nested PCR

SBT and cgMLST are common methods to genotype *L. pneumophila* isolates (Gaia et al., 2003; Moran-Gilad et al., 2015). In this study, we used *L. pneumophila*-specific nested PCR as a gold standard to evaluate the results of the metagenomic analysis. *Legionella* genus-specific nested PCR was conducted amplifying 16S rRNA genes using the outer primers Leg120v and Leg1023r (Buchbinder et al., 2002) and inner primers JFP and JRP (Cloud et al., 2000). *L. pneumophila*-specific nested PCR was conducted amplifying macrophage infectivity potentiator surface protein (*mip*) genes using the outer primers Lmip920 and Limp1548 (Mahbubani et al., 1990) and inner primers Lmip976 and Lmip1427 (Catalan et al., 1994). All PCR reactions were performed using Tks Gflex DNA Polymerase (TaKaRa Bio Inc., Shiga, Japan). The amplified PCR products were analyzed using agarose gel electrophoresis and visualized with a UV transilluminator. The amplicons of *mip* PCR were subjected to Sanger sequencing analysis. The obtained sequences were aligned using ClustalW (Larkin et al., 2007), and p-distances among sequences were calculated by MEGA6 (Tamura et al., 2013).

### Illumina Sequencing for Shotgun Metagenomic Analysis

The Illumina MiSeq platform was used for shotgun metagenomic analysis. The sequencing libraries were prepared with a Nextera XT DNA Sample Prep Kit (Illumina, San Diego, CA, United States). Libraries from each sample were tagged with multiplexing barcodes for analysis in one run. The final concentration of the purified libraries was normalized to 4 nM and the pooled libraries were sequenced with a MiSeq Reagent Kit v3 (Illumina). The resulting sequence data were made available at the DNA Data Bank of Japan (DDBJ) with an accession number of DRA006698. The barcoding sequences were removed using CLC Genomic Workbench software 8.0 (CLC bio, Tokyo, Japan). The resulting clean reads were used as shotgun reads for further analysis.

### GS Junior Sequencing for 16S rRNA Amplicon Analysis

The GS Junior Titanium System (Roche, Basel, Switzerland) was used for 16S rRNA amplicon analysis. The 16S rRNA library was prepared as described in the previous study (Qiu et al., 2014). The resulting sequence data were made available at the DDBJ with an accession number of DRA006697. Barcoding sequences were removed as described above and reads shorter than 250 bp were also removed using CLC Genomic Workbench software. Potential chimera sequences were removed using Chimera.Slayer (Haas et al., 2011).

### Taxonomic Classification of Reads From Shotgun Metagenomic and 16S rRNA Amplicon Analyses Using MEGAN

A blastn search (Altschul et al., 1990) and MEGAN (Huson et al., 2016) were used for taxonomic classification of the reads. For each sample, shotgun reads were aligned against the NCBI-NT database using blastn with a cut off value of 1e-04. Then, the blastn results were analyzed using the naïve LCA algorithm of MEGAN with parameters of min score = 50.0, max expected = 0.01, top percent = 10.0, min support percent = 0.001, and min support = 1. The proportions of bacterial genera (or species) were calculated using the numbers of reads classified to the genus (or species) divided by the numbers of reads classified as bacteria. Numbers of reads mapped to each bacterial genus in each sample were subjected to principal component analysis (PCA) using the prcomp command in R (R Core Team, 2016). The numbers of reads identified as *L. pneumophila* were collected after taxonomical classification. The reads generated from the 454 GS Junior Titanium System were aligned against the NCBI-16SMicrobial-NT database using blastn with a cut off value of 1e-04. The taxonomic classification and downstream analysis were conducted as mentioned above.

### Detection of *L. pneumophila* Using Kraken and CLARK

In addition to the analysis with MEGAN, we tested two *k*-mer-based taxonomic classification algorithms, Kraken (Wood and Salzberg, 2014) and CLARK (Ounit et al., 2015). For the Kraken analysis, the reference sequences (RefSeq) of bacteria, archaea, and viruses were downloaded from the Kraken webpage, and a standard Kraken database was constructed. Shotgun reads were aligned and classified to the bacterial taxonomy using Kraken v1.0 with default parameters. For CLARK, only the RefSeq of bacteria were obtained from the CLARK webpage, and they were used to construct a bacterial database. Shotgun reads of each sample were aligned and classified to the bacterial taxonomy using CLARK v1.2.3.2 with default parameters. In both analyses, the numbers of reads identified as *L. pneumophila* were collected after taxonomic classification.

### Detection of *L. pneumophila* Using Blastn Against VFDB

Nucleotide sequences of virulence factor genes were downloaded from the Virulence Factor Gene Database (VFDB) (Chen et al., 2005). A VFDB blast database was constructed using the ‘makeblastdb’ command in the blast package. Shotgun reads were aligned against the database using blastn with a cut off value of 1e-04. Blastn results with multiple hits from the same query to different regions of the same reference sequence were removed, except one. The proportions of *L. pneumophila* hits were calculated by dividing the number of reads classified to *L. pneumophila* by the number of reads classified to bacteria.

### Detection of *L. pneumophila* Using Blastn Against *mip* Gene

A nucleotide sequence of the *mip* gene from *L. pneumophila* subsp. *philadelphia* str. Philadelphia 1 (NC_002942.5) was download from NCBI, and a *mip* blast database was constructed using this sequence. Shotgun reads from each sample were aligned to this database using a blastn search with a cut off value of 1e-04, and the numbers of hit reads were collected.

### Detection of *L. pneumophila* Using Blastn Against a Custom VFDB

Based on the results of a blastn search of shotgun reads against a VFDB blast database, virulence factor genes (*n* = 9) associated with *L. pneumophila* were identified. For each virulence factor gene, its protein sequences of *L. pneumophila* subsp. *philadelphia* str. Philadelphia 1 were downloaded from NCBI. These protein sequences are CcmC (YP_094893.1), CcmF (YP_094896.1), DotA (YP_096691.1), IcmO (YP_094490.1), KatB (YP_096397.1), LvhB10 (YP_095278.1), PilT (YP_096029.1), GTP pyrophosphokinase (YP_095486.1), and superoxide dismutase (YP_096960.1). Nucleotide sequences encoding these nine proteins were collected using the tblastn search at NCBI (4,267, 4,526, 483, 707, 2,686, 5,506, 5,000, 5,000, and 5,000 sequences were obtained for *ccmC, ccmF, dotA, icmO, lvhB10, katB, pilT, relA*, and *sodB*, respectively) and a custom VFDB blast database was constructed. A blastn search of shotgun reads against the custom VFDB was performed, and the numbers of reads identified as *L. pneumophila* were obtained using the naïve LCA algorithm in MEGAN.

### Comparison of Database Search Methods for *L. pneumophila* Detection

The area under the curve (AUC) of receiver operating characteristic curve (ROC) was used to compare the results of different database search methods in the detection of *L. pneumophila*. We considered that the results of *L. pneumophila*-specific nested PCR were correct. The true positive rate and false positive rate (1 – specificity) of each database search method were calculated, and area under curves were determined using the AUC package (Ballings and Van den Poel, 2013) in R.

## Results

### Detection of *Legionella* spp. and *L. pneumophila* Using Nested PCR

**FIGURE 2:**
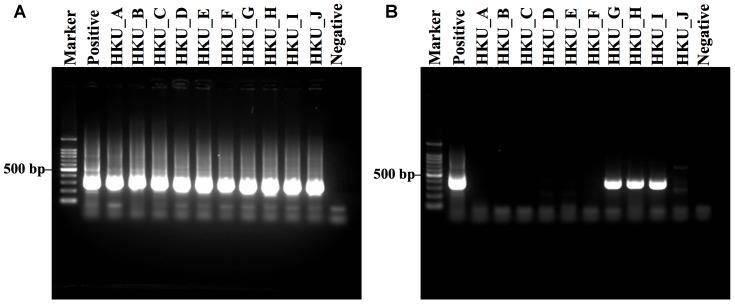
Gel electrophoresis of DNA amplification by *Legionella* genus-specific and *Legionella pneumophila***-**specific nested PCRs. **(A)** Amplification results of *Legionella* genus-specific and **(B)** *L. pneumophila*-specific nested PCRs are shown. Lane Marker: 100 bp DNA marker; lane Positive: *L. pneumophila*; lanes HKU_A – HKU_C and HKU_E – HKU_I: DNA from water samples of Sakushokotoni stream; lane HKU_D: DNA from water samples of Ohno Pond; lane HKU_J: DNA from water samples of Hyotan Pond; and lane Negative: distilled water (no DNA).

*Legionella* spp. was detected in all samples by *Legionella* genus-specific nested PCR (**Figure 2A**). The amplification of the *mip* gene by *L. pneumophila*-specific nested PCR was observed in only three samples; HKU_G, HKU_H, and HKU_I (**Figure 2B**). These results suggested that 7 samples, except for HKU_G, HKU_H, and HKU_I, contained *Legionella* spp. not classified as *L. pneumophila*. The pairwise distances among the *mip* gene sequences from amplified samples and positive control were within a range of 0.018 – 0.030, indicating that there was no cross contamination from the positive control during the PCR process. Therefore, we concluded that the samples HKU_G, HKU_H, and HKU_I were contaminated with *L. pneumophila*.

### Next Generation Sequencing

Next generation sequencing was conducted using Illumina MiSeq and GS Junior Titanium System, from which a total of 51,162,136 and 353,913 reads were obtained, respectively (**Table 1**). The average lengths of bacterial reads obtained from 16S rRNA amplicon analysis were within a range of 453.3 – 473.4 bp, whereas average lengths of bacterial reads obtained from Miseq were within a range of 288.8 – 293.1 bp.

**Table 1:** Summary of next generation sequencing reads.

Methods	Samples	Number of raw reads	Number of passed-QC reads	Number of reads hit with database	Number of reads identified as bacteria by MEGAN	Average length of bacterial reads
16S rRNA	HKU_A	46,968	39,245	39,166^a^	35,404	455.7
analysis	HKU_B	29,684	25,183	25,093^a^	24,711	453.3
	HKU_C	39,167	32,628	32,534^a^	32,256	450.5
	HKU_D	35,360	28,564	28,504^a^	28,409	461.5
	HKU_E	32,936	25,826	25,735^a^	25,654	464.9
	HKU_F	29,649	24,215	24,143^a^	24,063	465.4
	HKU_G	43,416	33,846	33,692^a^	33,636	466.8
	HKU_H	28,235	21,948	21,852^a^	21,735	462.8
	HKU_I	38,581	30,646	30,554^a^	30,510	468.5
	HKU_J	29,917	25,313	25,232^a^	25,065	473.4
Shotgun	HKU_A	1,554,614	N/A	318,064^b^	309,063	288.8
analysis	HKU_B	5,291,304	N/A	1,628,823^b^	1,600,198	293.1
	HKU_C	7,078,858	N/A	2,354,608^b^	2,323,879	289.7
	HKU_D	5,430,216	N/A	1,891,874^b^	1,873,938	284.5
	HKU_E	6,046,758	N/A	2,283,330^b^	2,264,076	285.4
	HKU_F	6,350,502	N/A	2,024,966^b^	2,006,002	284.7
	HKU_G	4,992,354	N/A	1,769,319^b^	1,752,738	285.8
	HKU_H	6,039,572	N/A	1,896,294^b^	1,872,777	286.3
	HKU_I	4,581,078	N/A	1,729,619^b^	1,714,434	285.5
	HKU_J	3,796,880	N/A	1,719,066^b^	1,710,319	289.7

*^a^Number of reads hit with the NCBI-16SMicrobial-NT database. ^b^Number of reads hit with the NCBI-NT database. N/A, Not applicable.*

### Bacterial Communities Inferred From 16S rRNA Amplicon and Shotgun Metagenomic Analyses

**FIGURE 3:**
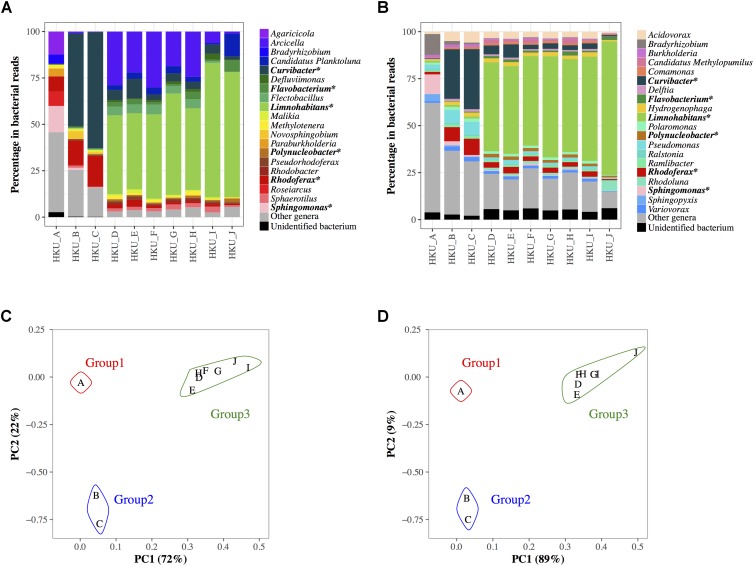
Bacterial communities at genus level in water samples determined by the lowest common ancestor algorithm in MEGAN. **(A)** Bacterial communities based on the results of a blastn search of 16S rRNA amplicon reads against nucleotide sequences from the NCBI-16SMicrobial-NT database. **(B)** Bacterial communities based on the results of the blastn search of shotgun sequencing reads against nucleotide sequences from the NCBI-NT database. Colored bars represent the top 20 abundant genera in all samples. Reads from other minor genera are represented in gray, and the reads with unidentified genera are represented in black. The genera ranked in top 20 in both 16S rRNA amplicon and shotgun metagenomic analyses are indicated with asterisks. The results of principal component analysis of the bacterial communities using 16S rRNA reads and shotgun reads are shown in **(C,D)**, respectively.

16S rRNA amplicon and shotgun sequence reads were subjected to a blastn search against NCBI-16SMicrobial-NT and NCBI-NT databases, respectively. The proportions of bacterial genera inferred using the naïve LCA algorithm of MEGAN are shown in **Figures 3A,B** for 16S rRNA amplicon and shotgun reads, respectively. More than 75% of reads generated by GS Junior Titanium System were identified as having bacterial origins, whereas 19.9 – 45.0% of Illumina reads were identified as having bacterial origins. A total of 977 bacterial genera were detected from 16S rRNA amplicon analysis, while a total of 897 bacterial genera were found in shotgun metagenomic analysis. The PCA suggested that the bacterial communities in samples were divided into three groups in both 16S rRNA amplicon and shotgun metagenomic analyses (**Figures 3C,D**).

Some genera showed similar proportions of reads between 16S rRNA amplicon and shotgun metagenomic analyses, whereas others did not. For example, more than 10% of reads were identified as *Sphingomonas* in both 16S rRNA amplicon and shotgun metagenomic analyses (14.1 and 10.4%, respectively) in group 1 (HKU_A). Shotgun metagenomic analysis identified *Pseudomonas* (6.6 – 7.0%) in group 2, but this genus was not found in the top 20 genera in the 16S rRNA amplicon analysis. The highest portion of a bacterium in group 3 was *Limnohabitans* in both the 16S rRNA amplicon and shotgun metagenomic analyses (41.0 – 72.4% and 47.3 – 71.1%, respectively). In contrast, the 16S rRNA amplicon analysis identified a moderate number of reads from *Arcicella* (1.1 – 30.2%) in group 3, but this abundant genus was not listed in the top 20 genera of the shotgun metagenomic analysis. Supplementary Table 1 shows the number of bacterial reads of species classified by MEGAN with NCBI-NT database. Supplementary Figure 1 presents potential pathogens at the species level identified by shotgun reads.

### Detection of *L. pneumophila* Using MEGAN, Kraken, CLARK, VFDB, and *mip* Gene

**FIGURE 4:**
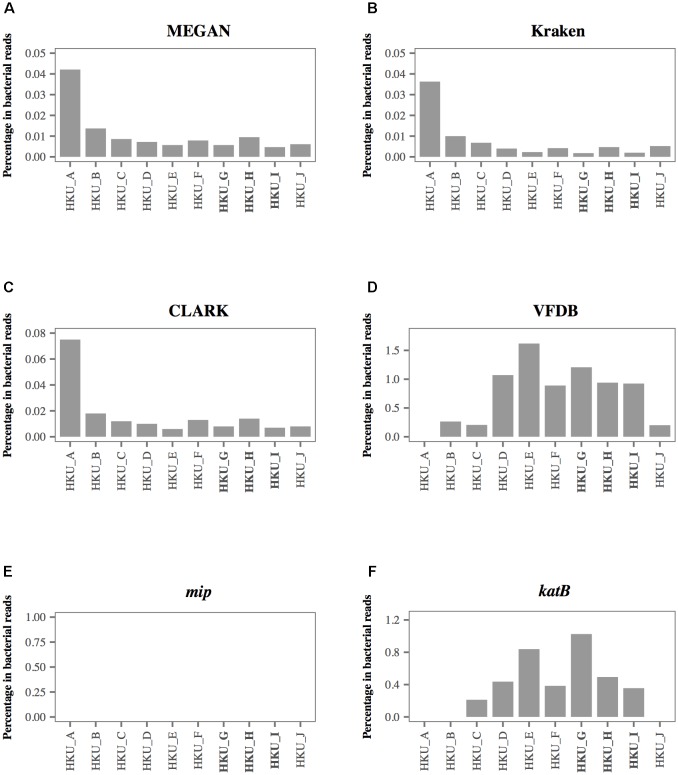
The percentage of shotgun reads identified as *Legionella pneumophila* using 6 different database-based search methods. Proportion of shotgun sequence reads identified as *L. pneumophila* by **(A)** MEGAN with the NCBI-NT database, **(B)** Kraken with RefSeq archaea, bacteria, and viruses, **(C)** CLARK with RefSeq archaea and bacteria, **(D)** VFDB, **(E)** *mip* gene, and **(F)** *katB* gene. The bold labels indicate *L. pneumophila*-positive samples using nested PCR.

To investigate the sensitivity and specificity of different database search methods in the detection of *L. pneumophila*, we compared the results of each method with that of *L. pneumophila*-specific nested PCR (**Table 2**). Although the nested PCR amplified sequence of the *mip* gene of *L. pneumophila* in three samples, blastn searches of shotgun reads could not detect any reads encoding the *mip* gene (**Table 2** and **Figure 4E**). In contrast, MEGAN with NCBI-NT database, Kraken and CLARK with RefSeq database detected a moderate number of *L. pneumophila* sequences in all samples (**Table 2**). MEGAN, Kraken, and CLARK identified the highest proportion of *L. pneumophila* reads in HKU_A (**Figures 4A–C**) even though HKU_A was negative by *L. pneumophila*-specific nested PCR assay. On the other hand, the use of VFDB detected no *L. pneumophila* read in HKU_A, and a relatively higher proportion of *L. pneumophila* reads in HKU_G, HKU_H, and HKU_I (**Figure 4D**), which were positive by nested PCR (**Figure 2B**). VFDB hits contained 19 virulence factor genes (Supplementary Table 2). Blastn searches of detected sequences against NCBI-NT indicated that 10 virulence factor genes were derived from other bacterial species. Finally, 9 virulence factor genes (*ccmC, ccmF, dotA, icmO, lvhB10, katB, pilT, relA*, and *sodB*) were identified as *L. pneumophila* origin (Supplementary Table 3).

**Table 2:** Number of shotgun reads identified as *Legionella pneumophila* by MEGAN, Kraken, CLARK, VFDB, and *mip* gene.

Samples	Nested PCR targeting the *mip* gene of *Legionella pneumophila*	Number of shotgun reads identified as *Legionella pneumophila*				
		
		MEGAN	Kraken	CLARK	VFDB	*mip* gene
HKU_A	Negative	130	90	99	0	0
HKU_B	Negative	220	136	125	19	0
HKU_C	Negative	200	134	117	22	0
HKU_D	Negative	135	63	63	27	0
HKU_E	Negative	129	45	45	59	0
HKU_F	Negative	159	71	83	28	0
HKU_G	Positive	100	27	42	28	0
HKU_H	Positive	178	75	79	24	0
HKU_I	Positive	81	30	40	18	0
HKU_J	Negative	104	86	34	3	0

### Detection of *L. pneumophila* Using a Custom VFDB

We further investigated the detection ability of the method using 9 virulence factor genes detected by the VFDB as a database. For each virulence factor gene, we collected related nucleotide sequences from its protein sequence using a tblastn search and constructed a custom VFDB. **Table 3** shows the number of shotgun reads identified as virulence factor genes associated with *L. pneumophila*. Among 9 genes we tested, the blastn search of shotgun reads against the *katB* gene of *L. pneumophila* showed the best agreement with the results of nested PCR.

**Table 3:** Number of shotgun reads identified as *Legionella pneumophila* using a blastn search against custom databases of virulence factor genes associated with *Legionella pneumophila*.

Samples	Number of reads identified as *Legionella pneumophila* / Number of reads identified as bacterial sequences								
	
	*ccmC*	*ccmF*	*dotA*	*icmO*	*lvhB10*	*katB*	*pilT*	*relA*	*sodB*
	(789 bp)^∗^	(1950 bp)^∗^	(3144 bp)^∗^	(2349 bp)^∗^	(1089 bp)^∗^	(2193 bp)^∗^	(1032 bp)^∗^	(2202 bp)^∗^	(588 bp)^∗^
HKU_A	0 / 123	0 / 193	0 / 0	0 / 0	1 / 120	0 / 226	0 / 176	0 / 71	0 / 77
HKU_B	2 / 172	0 / 317	2 / 4	0 / 18	1 / 113	0 / 733	2/ 1423	1 / 480	0 / 370
HKU_C	3 / 182	0 / 373	0 / 5	1 / 6	0 / 67	2 / 942	0 / 2440	0 / 742	0 / 529
HKU_D	0 / 279	0 / 595	0 / 0	0 / 0	0 / 10	2 / 458	0 / 637	0 / 452	0 / 603
HKU_E	1 / 386	0 / 704	0 / 0	0 / 0	0 / 15	5 / 596	0 / 1019	0 / 507	0 / 603
HKU_F	0 / 356	0 / 672	0 / 1	0 / 0	0 / 19	2 / 520	0 / 793	0 / 494	1 / 665
HKU_G	0 / 300	0 / 456	0 / 0	0 / 0	0 / 7	4 / 390	0 / 512	0 / 446	0 / 523
HKU_H	0 / 346	0 / 559	0 / 0	0 / 0	0 / 7	2 / 405	0 / 615	0 / 460	0 / 652
HKU_I	0 / 299	0 / 583	0 / 0	0 / 0	0 / 7	1 / 281	0 / 500	0 / 423	0 / 533
HKU_J	0 / 374	0 / 795	0 / 0	0 / 0	0 / 3	0 / 197	0 / 196	0 / 577	0 / 380

*^∗^Length of nucleotide sequence.*

### Diagnostic Ability of *L. pneumophila* Using a *katB* Gene

**Figure 4** presents the percentage of *L. pneumophila*-associated reads identified by 6 different database search methods. Among the 6 database search methods we tested, the blastn search against the *katB* gene showed the best agreement with the results of nested PCR. The highest percentage of shotgun reads identified as *L. pneumophila* origin was observed in HKU_G. The non-bacterial reads were classified as archaea, fungi, and metazoan reads. None of the reads identified as *L. pneumophila* was found in HKU_A, HKU_B, and HKU_J (**Figure 4F**).

**FIGURE 5:**
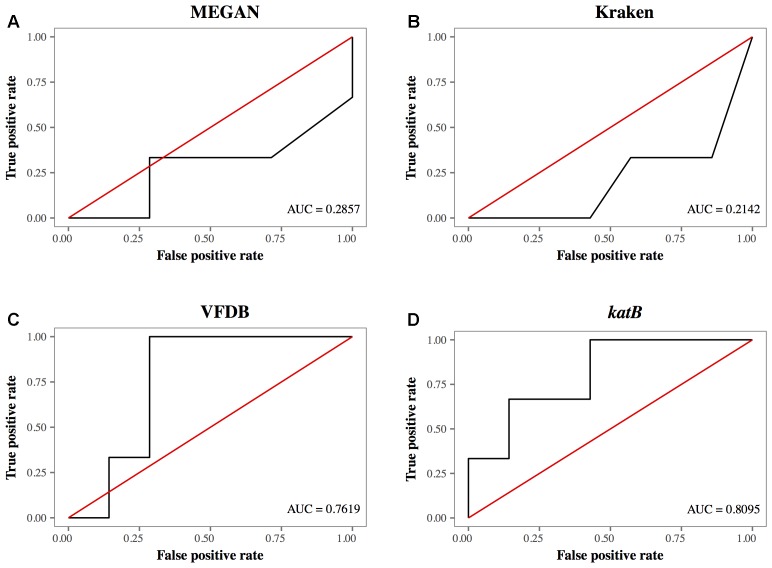
The receiver operating characteristic curves for different database search methods. **(A)** MEGAN with NCBI-NT database, **(B)** Kraken with RefSeq archaea, bacteria, and viruses, **(C)** VFDB, and **(D)** *kat*B gene. The red line is the reference line indicating the test without diagnostic benefit, i.e., random diagnosis.

The AUC of database search methods demonstrated that the detection of *L. pneumophila* using the *katB* gene had the highest AUC at 0.8095 (**Figure 5D**). Other database search methods such as MEGAN with NCBI-NT, Kraken and CLARK with RefSeq database had AUC values with a range between 0.2142 and 0.3095; lower than that using *katB* gene (**Figures 5A,B**). The database search method using the VFDB database had AUC value at 0.7619 (**Figure 5C**). These results indicate that the blastn search against the *katB* gene database had higher diagnostic capability than searches against databases containing whole genome sequences of *L. pneumophila*.

## Discussion

In this study, we conducted metagenomic analyses using water samples collected from a stream and ponds in the campus of Hokkaido University. By focusing on *L. pneumophila*, we evaluated different database search methods in detecting a specific bacterium in water samples by validating their detection results with those of nested PCR assay. We found that a blastn search of shotgun reads against the NCBI-NT database led to false positive detection and had a potential problem in specificity. Our results indicated that the blastn search against the genes of species-specific virulence factors had better agreement with the results of *L. pneumophila*-specific nested PCR.

The population structures inferred by 16S rRNA amplicon analysis and those by shotgun metagenomic analysis showed different bacterial communities even at the genus level (**Figures 3A,B**). On the other hand, PCA using 16S rRNA amplicon and shotgun metagenomic analyses clustered the samples in a similar way (**Figures 3C,D**). These results indicated that both 16S rRNA amplicon and shotgun metagenomic analyses captured the similarity in population structures among samples, but sensitivity and/or specificity of the two methods were different depending on bacterial genera.

The nested PCR assay detected *L. pneumophila* DNA in only three out of ten water samples (**Figure 2B**). In contrast, MEGAN with NCBI-NT database, Kraken and CLARK with the RefSeq database detected a moderate number of *L. pneumophila* sequences in the shotgun reads from all samples (**Table 2**). Furthermore, MEGAN with NCBI-NT, Kraken, and CLARK with RefSeq database detected a larger number of *L. pneumophila* sequences in PCR-negative samples such as HKU_B and HKU_C than in PCR-positive samples including HKU_G, HKU_H, and HKU_I (**Table 2**). Since the sensitivity of nested PCR assay with the employed primer sets is known to be 10 fg or 10 CFU per ml (Nintasen et al., 2007), the inconsistency is probably attributed to false positive detections due to the low specificity of these database search methods in detecting *L. pneumophila*.

The NCBI-NT and RefSeq databases contain whole genome sequences of *L. pneumophila*. The sequences of some of the bacterial genomic regions, for example the loci encoding housekeeping genes, are conserved among closely related bacterial species. The wrong assignment of the reads from such conserved genomic loci may be a possible cause of the false positive detection with MEGAN with NCBI-NT, Kraken and CLARK with RefSeq databases. In fact, the number of reads assigned to *L. pneumophila* were strongly correlated with the number of reads assigned to other species in genus *Legionella* with a Pearson correlation coefficient of 0.98 and a *p*-value of 10^-6^ (Supplementary Figure 2). A large fraction of reads assigned to *L. pneumophila* in HKU_A may be attributed to wrong assignment of reads from other abundant species in genus *Legionella* (**Figures 4A–C**).

The ROC plot analysis showed that detection using the *katB* gene had the largest AUC, indicating that the method was the best among the database search methods we tested (**Figure 5**). The *katB* gene can be found in several bacterial species, but nucleotide sequences of *katB* are divergent among different bacterial species (Supplementary Figure 3). This would be the reason for the high diagnostic ability of the method using the *katB* gene. The *mip* gene is a genetic marker for detecting *L. pneumophila* using PCR-based assay (Cianciotto et al., 1989). However, the shotgun reads did not contain a DNA fragment of the *mip* gene (**Table 2** and **Figure 4E**). The nucleotide length of the *mip* gene is 702 bp, while the length of a *katB* gene is 2,163 bp. The read depth of certain genes in shotgun metagenomic sequencing is proportional to the length of the gene. We speculate that the length of the *mip* gene might affect the absence of the gene in the metagenomic sequencing data. Despite *dotA* (3,144 bp) having more nucleotides than the *katB* gene, the number of reads identified as *L. pneumophila* using the *dotA* gene is smaller than that using the *katB* gene (**Table 3**). It is known that *dotA* determines the serogroup of *L. pneumophila* (Ko et al., 2003). There is a possibility that the *L. pneumophila* present in our samples belong to different serogroups from *L. pneumophila* subsp. *philadelphia* str. Philadelphia 1, which is the reference sequence we used for the tblastn search to collect nucleotide sequences.

The nested PCR using specific primers to amplify a *mip* gene detected *L. pneumophila* in only three samples; HKU_G, HKU_H, and HKU_I (**Figure 2B**). *L. pneumophila* can be found in natural water supplies (Mahbubani et al., 1990), and there is no report of outbreaks of *L. pneumophila* in the university campus. Since sampling the sites of HKU_G, HKU_H, and HKU_I are near a primeval forest conserved by the university, the pathogen has probably existed naturally and is not associated with the emergence of Legionnaires’ disease.

Although the detection of *L. pneumophila* using PCR-based methods is relatively rapid and sensitive, it is necessary to know the sequences of the target bacteria in advance. Conversely, a shotgun metagenomic approach does not require sequence information and thus is potentially useful in the detection of new and/or unexpected organisms. High throughput is another advantage of the metagenomic approach in that the method can detect multiple organisms in a single run. In fact, several studies have demonstrated the usefulness of metagenomic analysis in water science. Gomez-Alvarez et al. (2012) used metagenomics to investigate microbial populations in drinking water and found that *Legionella* like-genes were abundant in free-chlorine-treated drinking water. Metagenomic analysis showed potential risk of *Mycobacterium tuberculosis*-like in water samples from wastewater treatment plants (Cai and Zhang, 2013). Several studies have detected bacterial genes related to antibiotic resistance in water samples (Zhang et al., 2011; Durso et al., 2012; Wang et al., 2013). Pereira et al. (2017) proposed a novel approach to increase the sensitivity of *Legionella* detection in metagenomics. These studies are examples of possible directions for future application of metagenomics in detecting pathogens in water.

Our study has a limitation due to a lack of information for *L. pneumophila* in our water samples. The conventional method could be used to enumerate the number of *L. pneumophila* in a water sample. Based on the sensitivity the *L. pneumophila*-specific nested PCR (Nintasen et al., 2007), the number of *L. pneumophila* were estimated as at least 10 CFU/ml. Another limitation of this study was the number of reads generated by Miseq. Hiseq can produce a larger number of sequence reads with deeper coverage. In this sense, we might increase the sensitivity of detection of *L. pneumophila* by using Hiseq. At the same time, however, the length of reads from Hiseq are 100 – 150 bp, shorter than that of Miseq, which produces 300 bp. In this sense, specificity of detection might decrease if we used Hiseq. The number and the length of sequence reads are a tradeoff as well as sensitivity and specificity. These tradeoffs should be considered when conducting shotgun metagenomic analysis to detect pathogens in water samples. The one of our future work is the evaluation of detection limit of *L. pneumophila* in water samples using metagenomic analysis. Comparison of results among culture-based method, quantitative RT PCR, and metagenomic analysis can be used to discuss the detection limit of *L. pneumophila* in water samples.

In the present study, we compared the different database search methods for detecting *L. pneumophila* using metagenomic analyses. We used *L. pneumophila*-specific nested PCR as a gold standard and found that a blastn search against a *katB* gene database detected *L. pneumophila* with the highest area under the ROC among the tested search methods. Our study suggests that sequence searches targeting a long gene specifically associated with a bacterial species of interest has better diagnostic potential using current NGS technologies.

## Author Contributions

JB, RN, and KI designed the study. JB, RN, and CS conducted sampling and next generation sequencing. OS provided the positive control DNA of *L. pneumophila*. JB analyzed the data. JB, RO, and KI designed the statistical analysis and wrote the paper.

## Conflict of Interest Statement

The authors declare that the research was conducted in the absence of any commercial or financial relationships that could be construed as a potential conflict of interest.
