# Supplementary material for: Comparison of Database Search Methods for the Detection of Legionella pneumophila in Water Samples Using Metagenomic Analysis
Source: Front Microbiol. 2018 Jun 19;9:1272. doi: 10.3389/fmicb.2018.01272 (PMC6018159; doi:10.3389/fmicb.2018.01272)
Supplement: Supplementary file 2 [file Table_2.DOCX]

**Supplementary Table 2. Number of shotgun reads identified as virulence factor genes of *Legionella pneumophila* by blastn search against nucleotide sequences from Virulence Factor Gene Database**.

| **Genes** | **Samples and**  **(Numbers of shotgun reads identified as *Legionella pneumophila*)** | | | | | | | | | |
| --- | --- | --- | --- | --- | --- | --- | --- | --- | --- | --- |
|  | **HKU_A** | **HKU_B** | **HKU_C** | **HKU_D** | **HKU_E** | **HKU_F** | **HKU_G** | **HKU_H** | **HKU_I** | **HKU_J** |
|  | (865)* | (7,163)* | (10,682)* | (2,520)* | (3,645)* | (3,147)* | (2,320)* | (2,552)* | (1,949)* | (1,490)* |
| *ccmC* |  | 3 | 6 |  |  | 1 | 1 |  |  |  |
| *ccmF* |  |  | 1 |  | 1 |  |  |  |  |  |
| *dotA* |  | 2 |  |  |  |  |  |  |  |  |
| *fleQ* |  |  | 2 |  |  |  |  |  |  |  |
| *htpB* |  | 4 | 1 | 7 | 12 | 10 | 4 | 7 | 4 | 3 |
| *icm0* |  |  | 1 |  |  |  |  |  |  |  |
| *katA* |  | 1 |  |  |  |  |  |  |  |  |
| *katB* |  | 1 | 2 | 16 | 36 | 9 | 16 | 15 | 13 |  |
| *legK3* |  |  | 1 |  |  |  |  |  |  |  |
| *letS* |  | 2 | 2 | 4 | 8 | 3 | 2 | 1 |  |  |
| *lpg0773* |  |  |  |  |  | 1 |  |  |  |  |
| *lvhB10* |  | 1 |  |  |  |  |  |  |  |  |
| *motB* |  | 1 | 4 |  | 2 | 3 | 5 |  |  |  |
| *pgi* |  |  |  |  |  |  |  |  | 1 |  |
| *pilT* |  | 2 |  |  |  |  |  |  |  |  |
| *pilZ* |  |  |  |  |  |  |  | 1 |  |  |
| *relA* |  | 1 |  |  |  |  |  |  |  |  |
| *sdcB* |  |  | 2 |  |  |  |  |  |  |  |
| *sodB* |  | 1 |  |  |  | 1 |  |  |  |  |

* Number of shotgun reads hit to the database and identified as bacterial reads.
