# Supplementary material for: Comparison of Database Search Methods for the Detection of Legionella pneumophila in Water Samples Using Metagenomic Analysis
Source: Front Microbiol. 2018 Jun 19;9:1272. doi: 10.3389/fmicb.2018.01272 (PMC6018159; doi:10.3389/fmicb.2018.01272)
Supplement: Supplementary file 3 [file Table_3.DOCX]

**Supplementary Table 3. Number of shotgun reads identified as *Legionella pneumophila* genes and other bacteria.**

| **Genes** | **Species** | **Samples** | | | | | | | | | |
| --- | --- | --- | --- | --- | --- | --- | --- | --- | --- | --- | --- |
|  |  | **HKU_A** | **HKU_B** | **HKU_C** | **HKU_D** | **HKU_E** | **HKU_F** | **HKU_G** | **HKU_H** | **HKU_I** | **HKU_J** |
| *ccmC* | *Achromobacter xylosoxidans* |  |  |  |  |  | 1 |  |  |  |  |
|  | ***Legionella pneumophila*** |  | 2 | 3 |  |  |  |  |  |  |  |
|  | *Oceanisphaera profunda* |  | 1 |  |  |  |  |  |  |  |  |
|  | *Ralstonia pickettii* |  |  |  |  |  |  | 1 |  |  |  |
|  | *Sulfuricella denitrificans* |  |  | 2 |  |  |  |  |  |  |  |
| *ccmF* | *Achromobacter xylosoxidans* |  |  |  |  |  | 1 |  |  |  |  |
|  | ***Legionella pneumophila*** |  | 2 | 3 |  |  |  |  |  |  |  |
| *dotA* | ***Legionella pneumophila*** |  | 2 |  |  |  |  |  |  |  |  |
| *fleQ* | *Candidatus Midichloria mitochondrii* |  |  | 2 |  |  |  |  |  |  |  |
| *htpB* | *Acinetobacter johnsonii* |  |  | 1 |  |  |  |  |  |  |  |
|  | Beta proteobacterium |  |  |  | 1 |  |  |  | 1 |  |  |
|  | *Bdellovibrio bacteriovorus* |  |  |  |  |  | 1 |  |  |  |  |
|  | *Bdellovibrio exovorus* |  |  |  | 2 |  |  |  |  |  |  |
|  | *Bacillus* sp. |  | 1 |  |  |  |  |  |  |  |  |
|  | *Cellvibrio* sp. |  | 1 |  |  |  | 1 |  |  |  |  |
|  | *Legionella hackeliae* |  |  |  |  |  |  |  | 1 |  |  |
|  | *Methylotenera mobilis* |  | 1 |  |  |  |  |  |  |  |  |
|  | *Polynucleobacter duraquae* |  |  |  |  | 4 | 1 | 1 | 1 | 1 |  |
|  | *Polynucleobacter necessarius* |  |  |  | 4 | 8 | 7 | 3 | 4 | 3 | 3 |
| *icmO* | ***Legionella pneumophila*** |  |  | 1 |  |  |  |  |  |  |  |
| *katA* | *Dyella japonica* |  | 1 |  |  |  |  |  |  |  |  |
| *katB* | *Aeromonas salmonicida* |  |  |  | 4 | 6 | 1 | 5 | 4 | 2 |  |
|  | *Bacillus lehensis* |  |  |  |  |  |  |  | 1 |  |  |
|  | *Dechlorosoma suillum* |  |  | 1 |  |  |  |  |  | 2 |  |
|  | *Flavobacterium* sp. |  |  |  |  |  | 1 |  |  |  |  |
|  | *Hydrogenophaga* sp. |  |  |  |  | 2 |  |  |  |  |  |
|  | *Lacinutrix* sp. |  |  |  |  |  |  |  | 1 |  |  |
|  | *Laribacter hongkongensis* |  |  |  |  | 2 |  |  |  |  |  |
|  | ***Legionella pneumophila*** |  |  | 1 | 2 | 3 | 3 | 4 | 2 | 2 |  |
|  | *Marinovum algicola* |  |  |  | 3 | 2 |  |  |  | 2 |  |
|  | *Pseudomonas syringae* |  |  |  |  | 5 |  | 1 |  |  |  |
|  | *Vibrio owensii* |  | 1 |  |  |  |  |  |  |  |  |
|  | *Vitreoscilla filiformis* |  |  |  | 7 | 16 | 4 | 6 | 7 | 5 |  |
| *legK3* | No bacterium was identified |  |  |  |  |  |  |  |  |  |  |
| *letS* | *Caldilinea aerophila* |  |  |  |  |  | 1 |  |  |  |  |
|  | *Cellvibrio* sp. |  | 1 |  |  |  |  |  |  |  |  |
|  | *Curvibacter* sp*.* |  | 1 |  | 2 | 2 |  |  | 1 |  |  |
|  | *Geitlerinema* sp. |  |  | 1 |  |  |  |  |  |  |  |
|  | *Limnohabitans* sp. |  |  |  | 1 | 5 | 2 | 2 |  |  |  |
|  | *Pseudomonas* sp. |  |  |  |  | 1 |  |  |  |  |  |
|  | *Rhodoferax saidenbachensis* |  |  |  | 1 |  |  |  |  |  |  |
|  | *Vitreoscilla filiformis* |  |  | 1 |  |  |  |  |  |  |  |
| *lpg0773* | No bacterium was identified |  |  |  |  |  |  |  |  |  |  |
| *lvhB10* | ***Legionella pneumophila*** |  | 1 |  |  |  |  |  |  |  |  |
| *motB* | *Curvibacter* sp*.* |  |  | 1 |  | 1 |  | 1 |  |  |  |
|  | *Curvibacter* sp. |  | 1 | 3 |  | 1 | 3 | 4 |  |  |  |
| *pgi* | *Polynucleobacter necessarius* |  |  |  |  |  |  |  |  | 1 |  |
| *pilT* | ***Legionella pneumophila*** |  | 2 |  |  |  |  |  |  |  |  |
| *pilZ* | No bacteria was identified |  |  |  |  |  |  |  |  |  |  |
| *relA* | ***Legionella pneumophila*** |  | 1 |  |  |  |  |  |  |  |  |
| *sdcB* | *Legionella longbeachae* |  |  | 2 |  |  |  |  |  |  |  |
| *sodB* | *Glaciecola psychrophila* |  | 1 |  |  |  |  |  |  |  |  |
|  | ***Legionella pneumophila*** |  |  |  |  |  | 1 |  |  |  |  |
