# Supplementary material for: Comparison of Database Search Methods for the Detection of Legionella pneumophila in Water Samples Using Metagenomic Analysis
Source: Front Microbiol. 2018 Jun 19;9:1272. doi: 10.3389/fmicb.2018.01272 (PMC6018159; doi:10.3389/fmicb.2018.01272)

Supplementary Figure 2. Scatter plot comparing numbers of shotgun reads identified as *Legionella pneumophila* and *Legionella* spp. using MEGAN with NCBI-NT.

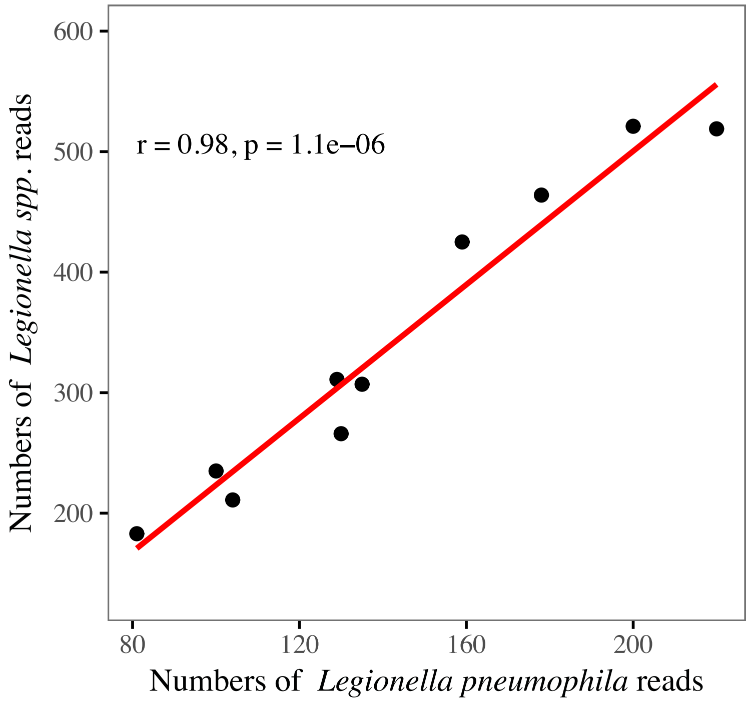

Supplement: Supplementary file 5 [file Image_2.PDF]
