# Supplementary material for: Comparison of Database Search Methods for the Detection of Legionella pneumophila in Water Samples Using Metagenomic Analysis
Source: Front Microbiol. 2018 Jun 19;9:1272. doi: 10.3389/fmicb.2018.01272 (PMC6018159; doi:10.3389/fmicb.2018.01272)

Supplementary Figure 3. Phylogenetic tree of catalase-peroxidase sequences from *Legionella pneumophila* and other bacteria. The tree was constructed using a maximum likelihood function in MEGA6 based on the nucleotide sequences of genes. The bootstrap confidence values were generated using 1,000 permutations. Different symbols indicate different genes from L. pneumophila: triangle for *katB* and circle for *katG*.

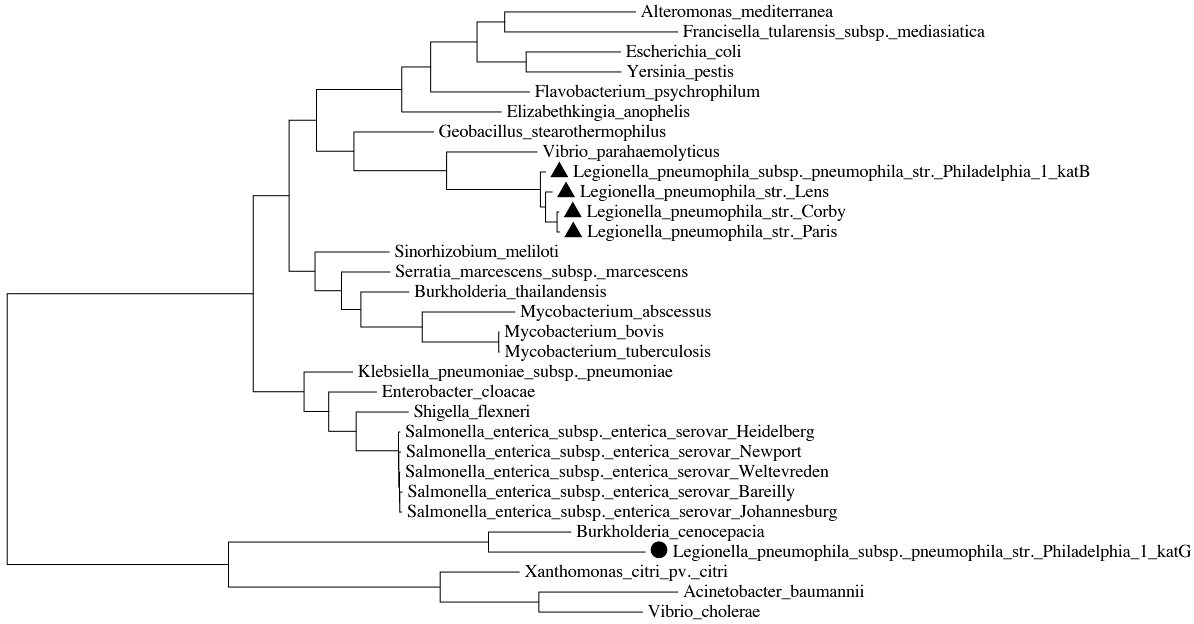

Supplement: Supplementary file 6 [file Image_3.PDF]
