# Supplementary material for: Comparison of Database Search Methods for the Detection of Legionella pneumophila in Water Samples Using Metagenomic Analysis
Source: Front Microbiol. 2018 Jun 19;9:1272. doi: 10.3389/fmicb.2018.01272 (PMC6018159; doi:10.3389/fmicb.2018.01272)

Supplementary Figure 1. Heatmap of potential pathogens identified by MEGAN using NCBI-NT from shotgun reads. Detected bacteria are shown in grey, which refers to reads higher than 0.001%. Undetected bacteria are shown in white. The results may contain false positives.

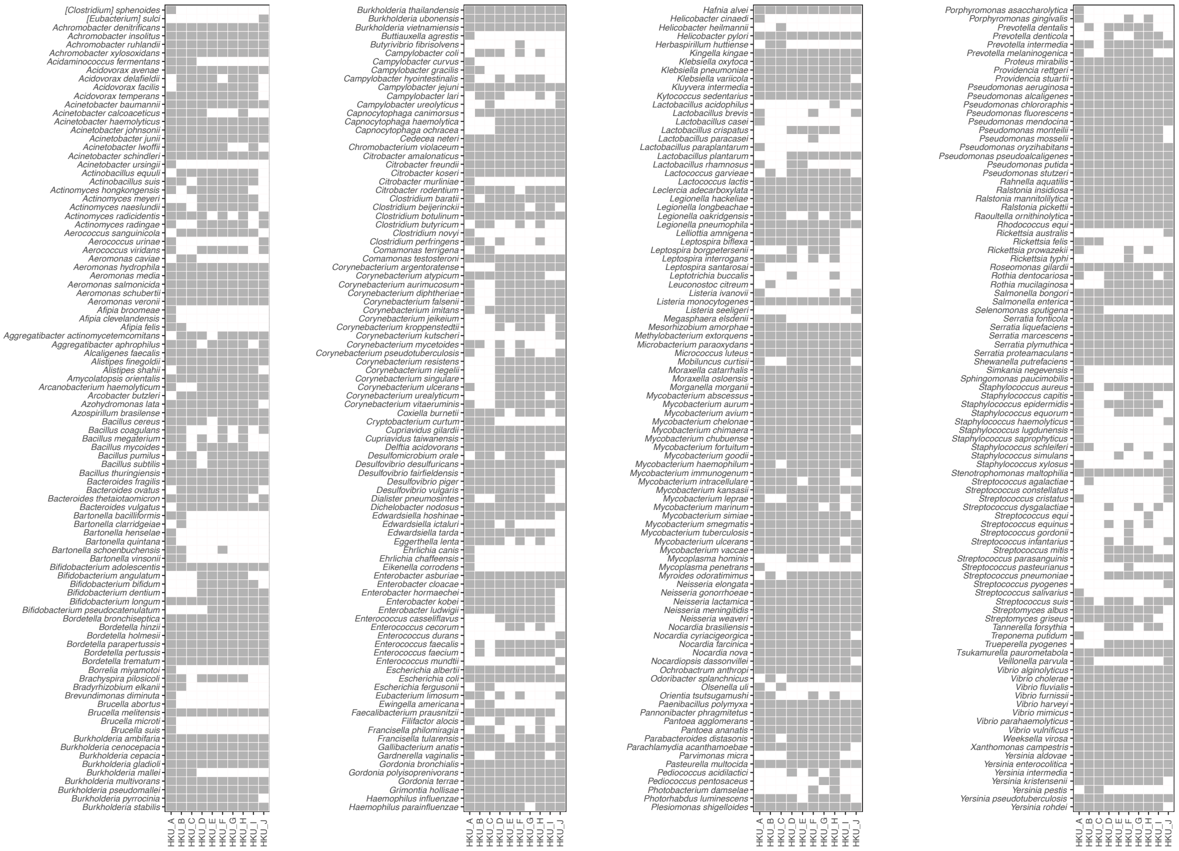

Supplement: Supplementary file 4 [file Image_1.PDF]
